# Bismuth Quantum Dots in Annealed GaAsBi/AlAs Quantum Wells

**DOI:** 10.1186/s11671-017-2205-7

**Published:** 2017-06-30

**Authors:** Renata Butkutė, Gediminas Niaura, Evelina Pozingytė, Bronislovas Čechavičius, Algirdas Selskis, Martynas Skapas, Vytautas Karpus, Arūnas Krotkus

**Affiliations:** Center for Physical Sciences and Technology, Saulėtekio ave. 3, LT-10257 Vilnius, Lithuania

**Keywords:** Bismides, Quantum nanostructures, Molecular beam epitaxy, High-resolution transmission electron microscopy, Raman spectroscopy, Photoluminescence

## Abstract

Formation of bismuth nanocrystals in GaAsBi layers grown by molecular beam epitaxy at 330 °C substrate temperature and post-growth annealed at 750 °C is reported. Superlattices containing alternating 10 nm-thick GaAsBi and AlAs layers were grown on semi-insulating GaAs substrate. AlAs layers have served as diffusion barriers for Bi atoms, and the size of the nanoclusters which nucleated after sample annealing was correlating with the thickness of the bismide layers. Energy-dispersive spectroscopy and Raman scattering measurements have evidenced that the nanoparticles predominantly constituted from Bi atoms. Strong photoluminescence signal with photon wavelengths ranging from 1.3 to 1.7 μm was observed after annealing; its amplitude was scaling-up with the increased number of the GaAsBi layers. The observed photoluminescence band can be due to emission from Bi nanocrystals. The carried out theoretical estimates support the assumption. They show that due to the quantum size effect, the Bi nanoparticles experience a transition to the direct-bandgap semiconducting state.

## Background

GaAsBi-based heterostructures have a large potential for optoelectronic applications in a wide spectral range extending from near- to mid-infrared region. GaAsBi is a group III–V semiconductor compound that is actively investigated for GaAs-based infrared radiation emitters [1] and detectors [2–5]. Light-emitting diodes with GaAsBi active layers radiating at the wavelengths of ~987 nm (at Bi content of 1.8%) were described by Lewis et al. [6]; the electrically injected bismide laser with ~6% Bi in a GaAsBi/GaAs multi-quantum well (MQW) was reported in Ref. [7]. The main difficulty in this field is an increase of non-radiative recombination center density due to the low substrate temperatures needed for a molecular beam epitaxy (MBE) growth of GaAsBi layers with Bi content above 5%. One of the standard technological procedures allowing for a reduction of non-radiative recombination rate is a post-growth annealing at temperatures higher than those used for MBE growth. However, in the case of GaAsBi, the effect of annealing is not unambiguous. It has been shown previously by our group [8, 9] that the annealing at temperatures above 600 °C leads to several new features, the most non-trivial of which is an onset in some of the samples of rather intense photoluminescence (PL) in the wavelength range from 1.35 to 1.5 μm, this process being accompanied by substantial changes in GaAsBi epitaxial layer—a reduction of Bi content in the crystalline lattice and an appearance of nanometer-size clusters [9].

The growth of nanostructures—nanowires, strained quantum wells or quantum dots (QDs)—is a popular way to obviate the lattice mismatch between a substrate and the epitaxial layer grown on it. The most widely studied examples of QDs based on III–V compounds are the InGaAs- [10] and InGaN- [11] based QDs grown by Stranski–Krastanow technique [12]. In the case of GaAsBi, such a growth mechanism is still not realized. The nucleation of Bi-related clusters in annealed epitaxial GaAsBi layers and their structural characteristics has been systematically studied in Ref. [13]. It has been shown that nanoclusters of different crystalline structures and compositions—rhombohedral As and Bi as well as zinc-blende GaAsBi nanoclusters—are nucleating in the bismide layers upon annealing; their size is varying between 5 and 20 nm. Rhombohedral clusters of pure bismuth were observed in GaAsBi with relatively large Bi content (4.7%) grown at low temperature (200 °C) [13]. In our previous study [9], a formation of nanometer-size Bi clusters was reported in the high-temperature-annealed GaAsBi with Bi content above 6%. Moreover, it has been assumed that due to the size quantization effects, the bismuth nanoclusters become semiconducting rather than semi-metallic (as it is the case in the bulk Bi crystals), and that, the radiative recombination taking place in Bi clusters can be responsible for the long wavelength emission observed in annealed GaAsBi samples [9].

The present work reports on a formation of Bi nanocrystals in annealed GaAsBi/AlAs quantum wells. The AlAs layers were serving as the barriers both for a charge carrier confinement and for preventing Bi out-diffusion from GaAsBi layers during an annealing procedure. Presence of AlAs layers has secured a nucleation of Bi nanoparticles in a more controlled way—their size distribution was narrower and their density was higher than in annealed bulk layers [9]. The samples obtained were characterized by high-resolution transmission electron microscopy (HRTEM), PL and Raman spectroscopy measurements. Results of these experiments indicate a presence of pure Bi nanocrystals in the annealed heterostructures. Theoretical estimates performed confirm that Bi nanocrystals can be transformed by the size quantization effects to the direct gap semiconductors.

## Methods

GaAsBi/AlAs MQW structures were grown on semi-insulating GaAs (100) substrates using SVT-A MBE reactor equipped with metallic Ga, Al, and Bi sources as well as a two-zone cracker source to produce As_2_. The following MBE growth scheme was used. Firstly, the GaAs buffer layer (of about 100 nm) and the first AlAs barrier were grown using the standard MBE growth mode at the high temperature of 600 °C. Then, the growth was interrupted and the substrate temperature was lowered for a growth of GaAsBi QWs and AlAs barriers. Migration-enhanced epitaxy (MEE) mode was used for AlAs deposition at the following growth sequence: one monolayer (ML) of Al, 5 s interruption for a migration of group III atoms, then a supply of 1 ML of As [14, 15]. Finally, the MQW structure was covered by 5 nm-thick GaAs capping layer. The content of Bi in GaAsBi layers was determined from the (200)-reflex of ω-2Θ XRD scan and was about 7% for the as-grown samples.

Two different MQW samples were chosen for measurements. The MQW A-sample contains three 10 nm-thick and one 20 nm-thick GaAsBi QWs (MBE-grown at 330 °C) separated by 20 nm-thick AlAs barriers (MEE-grown at the same temperature). The MQW B-sample contains 20 QWs with 10 nm-wide GaAsBi layers separated by 4 nm-thick AlAs barriers grown under similar conditions as those used for growth of the A-sample. The high-temperature treatment of both samples was carried out in the rapid thermal annealing (RTA) oven at the temperature of 750 °C for 180 s at nitrogen ambient. To prevent arsenic loss from the surface layer, while annealing, the samples were covered by a sacrificial GaAs wafer.

The atomic force microscopy surface analysis demonstrated droplet-free surfaces of both as-grown and annealed MQW structures. The surface roughness of the GaAs cap layer was below 1 nm. The structural high-resolution measurements of nanoparticles, which were formed in MQWs after sample annealing, were carried out by FEI Tecnai G2 F20 X-TWIN TEM with STEM module, equipped with an X-ray energy-dispersive spectroscopy (EDS) detector for elemental mapping and a high-angle annular dark-field (HAADF) detector for Z-contrast imaging. FEI Helios Nanolab 650 dual beam microscope equipped with an Omniprobe manipulator was used to prepare specimens for the TEM measurements.

**Fig. 1:**
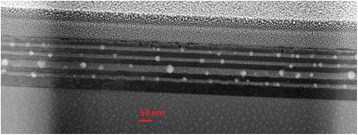
STEM image of the A-sample with three 10 nm-thick and one 20 nm-thick GaAsBi QWs grown by MBE and 20 nm-thick MEE-grown AlAs barriers after annealing at 750 °C temperature for 180 s

**Fig. 2:**
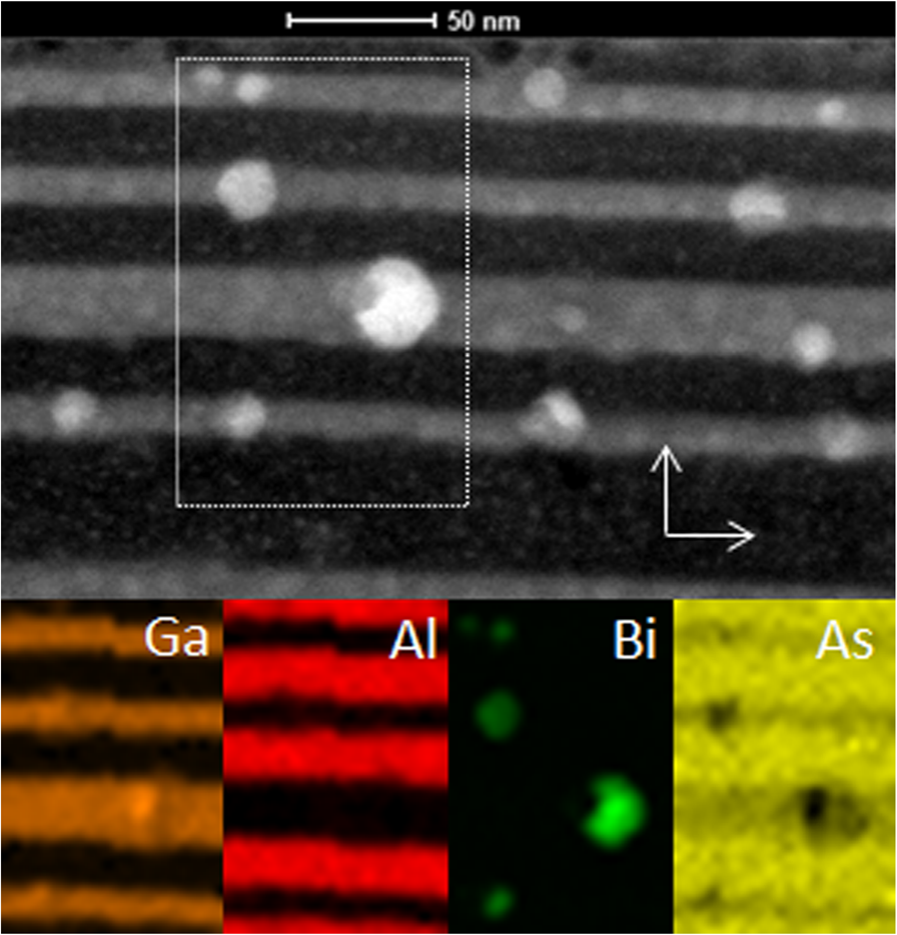
HAADF–STEM Z-contrast image of Bi nanocrystals in GaAsBi/AlAs MQW structures (*above*). The EDS images (*below*) represent the elemental mapping of intensities of Ga, Al, Bi, and As, measured on the marked area of STEM image

Figure 1 shows STEM image of the A-sample. The image evidently reveals numerous nanoparticles, which were formed in GaAsBi quantum-well (QW) layers after annealing. An obvious correlation between the size of nanoparticles and the width of QW layers can be traced in the image. The correlation evidences that AlAs layers (darkest regions) are effectively acting as the barriers preventing an out-diffusion of Bi atoms from GaAsBi layers. The EDS elemental mapping of selected area of the sample simultaneously obtained with HAADF imaging shows (Fig. 2) that the formed nanocrystals are predominantly constituted of bismuth atoms.

## Results

### Raman Spectroscopy

The Raman spectra of the investigated GaAsBi MQW samples were recorded in the backscattering geometry by Via Raman (Renishaw) spectrometer equipped with a thermoelectrically cooled (−70 °C) CCD camera and a microscope. The 532-nm radiation line from diode-pumped solid-state laser was used for a photoexcitation. The 50×/0.75 NA objective lens and 1800 lines/mm grating were used to record the Raman spectra. The accumulation time was 400 s. To avoid the sample damage, the laser power at the sample was restricted to 0.06 mW. The Raman frequencies were calibrated using the silicon standard (line at 520.7 cm^−1^). Parameters of the vibrational modes were determined by fitting the experimental spectra with Gaussian–Lorentzian shape components using GRAMS/A1 8.0 (Thermo Scientific) software.

**Fig. 3:**
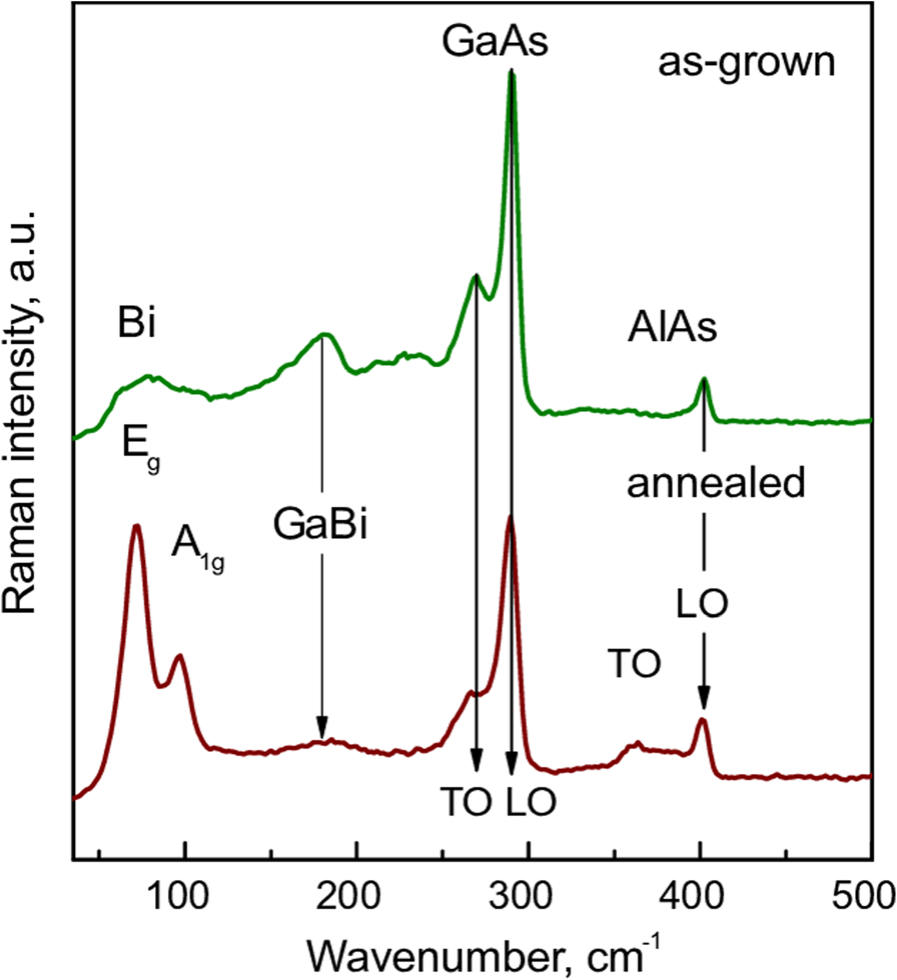
Raman spectra of the as-grown (*green curve*) and annealed (*red curve*) GaAsBi MQW A-sample

The Raman spectra of the as-grown and annealed GaAsBi/AlAs MQW A-sample are presented in Fig. 3. An intense doublet observed in the as-grown sample (Fig. 3, green curve) at 269 and 290 cm^−1^ corresponds to the GaAs-like transverse optical (TO) and longitudinal optical (LO) phonon modes, respectively [16–18]. In the backscattering geometry, the TO band is symmetrically forbidden for the ideal GaAs crystal [17, 18], but Bi-induced crystalline structure disorder breaks the symmetry of GaAs crystalline lattice and activates TO mode. Two other broad Bi-induced vibrational modes visible near 227 and 181 cm^−1^ can be attributed to GaBi-like vibrational modes [18]. The presence of AlAs barriers can be recognized in the Raman spectrum from a sharp LO mode at 402 cm^−1^ [19].

Relatively short (180 s) thermal annealing of the sample at 750 °C induces essential changes in the Raman spectrum: (i) intense low frequency bands appear at 72 and 96 cm^−1^, (ii) intensity of the bands near 269, 227, and 181 cm^−1^ decreases, and (iii) a broad feature near 361 cm^−1^ appears in the annealed sample spectrum. The two low-frequency bands at 72 and 96 cm^−1^ correspond well to *E*_g_ and *A*_1g_ modes of the crystalline bismuth [20–24]. The appearance of these bands together with a decrease in intensity of the Bi-induced GaBi-like bands at 269 and 181 cm^−1^ shows that thermal annealing causes in withdrawing of bismuth from the GaAsBi lattice sites and its agglomeration to Bi nanocrystals. Moreover, the formation of bismuth nanocrystals also affects the crystalline structure of AlAs layers, as it is apparent from the rise of a broad defect-induced TO feature near 361 cm^−1^ [25].

### Photoluminescence Measurements

The temperature-dependent photoluminescence (PL) measurements were carried out using a 500-mm focal length monochromator (Andor SR-500i) along with the liquid nitrogen cooled InGaAs photodetector. A diode-pumped solid-state laser emitting at the wavelength of 532 nm was used as an excitation source at the excitation power of 38 mW. The samples were mounted on the cold finger of a closed-cycle helium cryostat coupled with temperature controller, allowing for measurements in the temperature range of 3–300 K.

**Fig. 4:**
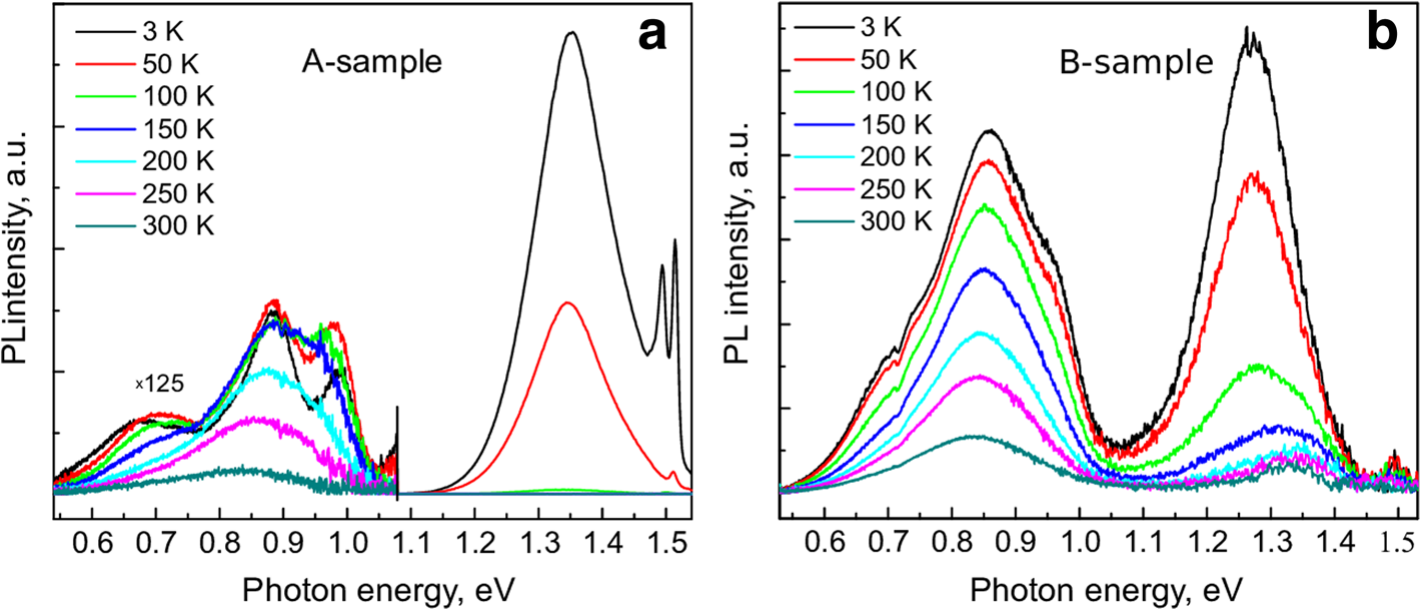
Temperature-dependent PL spectra of the annealed **a** A-sample composed of three 10 nm-wide and one 20 nm-wide GaAsBi/AlAs QWs and **b** B-sample composed of 20 10 nm-wide GaAsBi/AlAs QWs

The PL spectra of the annealed A-sample, which contains three 10 nm-thick and one 20 nm-thick GaAsBi QWs, measured at different temperatures are presented in Fig. 4a. Two major sets of spectral features below the bandgap of GaAs can be distinguished. Strong higher-energy peak situated at about 1.35 eV can be attributed to radiative transitions in GaAsBi QWs. Position of the peak is close to that observed in GaAs_0.979_Bi_0.021_/GaAs quantum wells [26] and correlates with XRD data which indicated the Bi content of 2.1% in the GaAsBi QW layers of A-sample after its annealing. The spectral features at low-energy side, 0.6–1.05 eV, appear in PL spectra after a thermal annealing of the sample and, therefore, can be attributed to optical transitions in Bi nanocrystals. The low-energy PL band has an inner structure, which reveals itself at low temperatures. Namely, at *T* = 3 K, the PL components positioned at 0.67, 0.88, and 0.98 eV can be distinguished. As it is seen from Fig. 4a, at liquid helium temperature, the PL signal from GaAsBi QWs is two orders of magnitude stronger than the low-energy PL band. However, the high-energy PL peak decreases rapidly with an increase of temperature and the low-energy PL peak starts to dominate at *T* > 100 K.

**Fig. 5:**
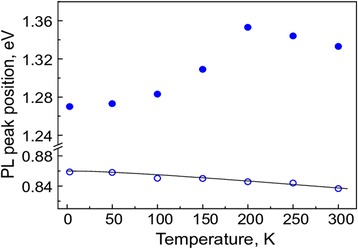
Temperature dependencies of spectral positions of the high- and low-energy PL bands for the annealed B-sample composed of twenty 10 nm-wide GaAsBi/AlAs QWs

The PL spectra of B-sample, which contains twenty 10 nm-thick GaAsBi quantum wells separated by 4 nm-thick AlAs barriers, are presented in Fig. 4b. Prior to thermal annealing, as was the case for the A-sample, the B-sample manifested only the higher-energy, QW-related, PL band. The strong low-energy PL peak at about 0.85 eV was observed after the thermal annealing and, therefore, we assume, can be attributed to emission from Bi nanocrystals. Intensity of the low-energy peak in B-sample is stronger than that in A-sample and scales up with an increased number of QWs. At low temperatures, three components of the peak, which were well-resolved in A-sample, can be traced. However, in the B-sample, the low-energy PL peak is dominated by its 0.85 eV component in the whole temperature range investigated. Position of the higher-energy, QW-related, PL peak is slightly shifted to lower energies with respect to its position in A-sample in accordance with XRD data, which indicated the 2.8% Bi in quantum-well layers of B-sample after its thermal annealing. In B-sample, the QW-related PL peak reveals its inner structure. The peak is constituted from the bound exciton related component at about 1.27 eV, which dominates at low temperatures, and delocalized exciton related component, which is situated at about 1.35 eV and is dominating at higher-temperatures. The inner structure of the QW-related peak results in a characteristic S-type temperature dependence of the PL peak position (full dots in Fig. 5), which was observed previously both in bulk GaAsBi [27] and in GaAsBi/GaAs quantum wells [26]. The PL peak positioned at low photon energies shows much weaker temperature dependence (open dots and curve in Fig. 5), which can be fitted by the Varshni function *E*(*T*) = *E*(0) − *αT* ^2^/(*β* + *T*) with the α and β parameters equal to 10^−4^ eV/deg and 100 K, respectively. It should be noted that the value of α parameter, responsible for the energy gap variation with temperature, is much smaller than its standard values for a majority of semiconductors, 3°10^−4^–5°10^−4^ eV/deg. This makes Bi nanocrystal matrix an important potential system for the light sources emitting at telecom wavelengths and having low temperature sensitivity.

## Discussion

The HRTEM, EDS, and Raman spectroscopy measurements carried out in the present study indicate that Bi nanocrystals (quantum dots) precipitate in GaAsBi layers after a thermal annealing of the low-temperature MBE-grown GaAsBi/AlAs MQW samples. One can assume that these nanocrystals are responsible for the long-wavelength photoluminescence band which reveals itself in the annealed samples. Although the bulk bismuth is semi-metallic, the small effective masses of Bi charge carriers result in an early onset of quantum confinement effects in Bi-based nanostructures. In fact, one of the first experimental observations of the size-quantization effects was reported for thin Bi layers [28]. Semimetal-to-semiconductor transition in thin Bi films, *d* < 30 nm, was experimentally observed in Ref. [29]. The transition has been also revealed in Bi nanowires with diameters smaller than 65 nm [30, 31]. In both these cases, the semiconducting state was identified from measurements of the temperature-dependent electric characteristics. The quantum size effect in bismuth nanoparticles was for the first time studied by electron energy loss spectroscopy [32], and the semi-metal to semiconductor transition was found to occur in Bi nanoparticles with diameters below 40 nm. The transition to the direct semiconductor state was lately reported [33] for colloidal 3.3 nm Bi nanoparticles.

In pure Bi, the principle valleys of electrons and holes are located at the *L* and *T* points of the Brillouin zone and correspond to ellipsoidal isoenergetic surfaces (Table 1). The ground state of the ellipsoidal valley electrons (holes) in a spherical quantum dot can be approximately estimated as

**Table 1:** Parameters of the bulk Bi energy structure [35–37] (*m*_0_ is the free electron mass)

Symmetry point	*E*_g_	Fermi energy	Effective mass components	$\overline{m}$
*T*	0.407 eV	11.6 meV	*m*_1_ = *m*_2_ = 0.059 *m*_0_	$\overline{m} = 0.0846\;{m}_0$
			*m*_3_ = 0.634 *m*_0_	
*L*	0.015 eV	26.6 meV	*m*_1_ = 0.00521 *m*_0_	$\overline{m} = 0.0113\;{m}_0$
			*m*_2_ = 1.21 *m*_0_	
			*m*_3_ = 0.0136 *m*_0_	

1 $$ W=\frac{\pi^2{\hslash}^2}{2\overline{m}{r}_0^2}\ . $$

Here *r*_0_ is the QD radius and $\overline{m}$ is the average inverse effective mass,

2 $$ \frac{1}{\overline{m}}=\frac{1}{3}\left(\frac{1}{m_1}+\frac{1}{m_2}+\frac{1}{m_3}\right), $$

*m*_1_, *m*_2_, and *m*_3_ are the principal effective masses of the ellipsoidal valley.

The phenomenological formula (1) gives a close estimate of the ground energy level ε_1_ in an infinitely deep spherical QD at arbitrary ratios of the effective masses. Indeed, it is exact, ε_1_ = *W*, in the case of a spherical isoenergetic surface (*m*_1_ = *m*_2_ = *m*_3_), predicts the ε_1_ energy with an accuracy of 12%, ε_1_ ≈ 0.88 *W*, and 25%, ε_1_ = 0.75 *W*, in the limiting cases of strongly prolate spheroidal valley (*m*_1_ = *m*_2_, *m*_3_ → ∞) and strongly oblate spheroidal one (*m*_1_ = *m*_2_, *m*_1_ → ∞), respectively. Therefore, at arbitrary values of the principle effective masses, formula (1) approximates the QD ground energy with accuracy better than 25%.

**Fig. 6:**
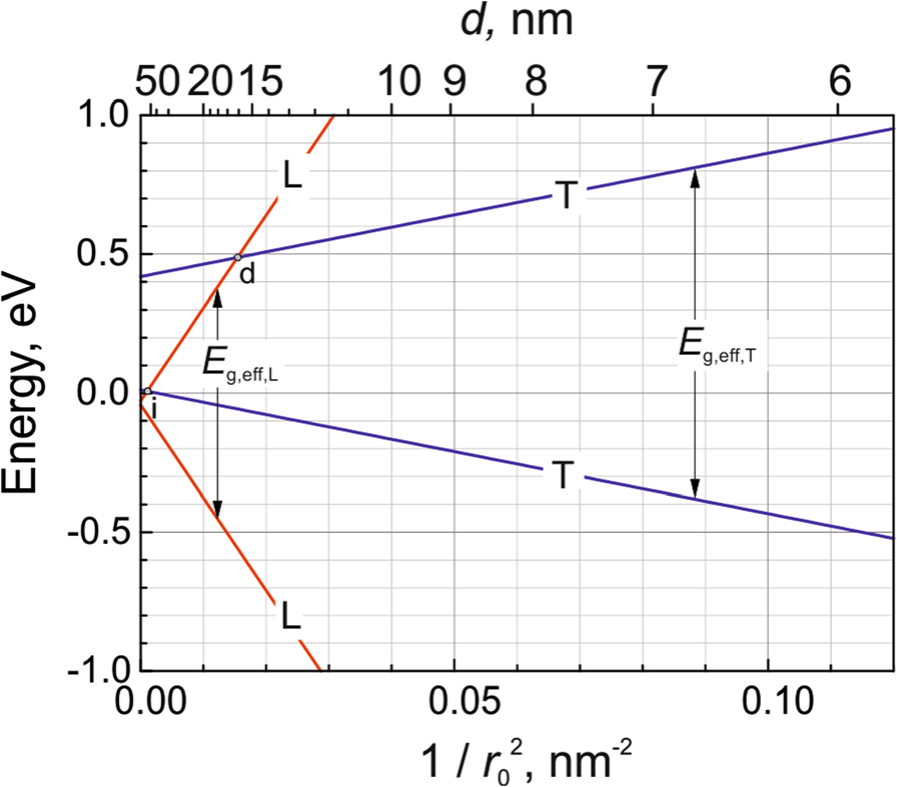
Evolution with the Bi QD energy spectrum with a decrease of its size (*r*_0_ and *d* are the QD radius and diameter)

Formula (1) allows for a simple straightforward evaluation of the effective energy gaps in bismuth quantum dots, *E*_g,eff_ = *E*_g_ + *W*_e_ + *W*_h_, where *E*_g_ is an energy gap in a bulk crystal and *W*_e_ and *W*_h_ are the electron and hole size quantization energies (1). The calculated effective *T* and *L* energy gaps are presented graphically in Fig. 6. (The electron and hole masses at both *T* and *L* points were assumed to be equal.)

In the bulk, semi-metallic bismuth, the conduction band minimum of the *L* valley is 38 meV below the *T* valence band maximum. When a size of Bi particles is reduced, the effective energy bandgap at the *L* point increases faster than that at the *T* point due to smaller effective masses of the *L*-valley, what is eventually leading to the semimetal to semiconductor transition (the *i*-crossover point in Fig. 6). At first, a bismuth nanocrystal becomes the indirect semiconductor with the lowest conduction band minimum at the *L* point and the highest valence band maximum at the *T* point. With a further decrease of the QD size, both the valence and conduction band edges will emerge at the *T* points making the Bi QD to become a direct gap semiconductor (the *d*-crossover point in Fig. 6).

It should be noted that Fig. 6 presents only a rough scheme of the energy spectrum, because the scheme disregards the non-parabolicity effects and assumes the infinite energy barriers for QDs. A deviation from the parabolic dispersion law is essential for the *L*-valley (see e.g., [34]). Indeed, the effective masses at the *L*-valley center are approximately five times smaller than their values at the Fermi energy (which were used for calculations of the energy spectrum presented in Fig. 6). On the other hand, the non-parabolicity effects are weaker at the *T* points, where the energy bandgap is larger, and therefore, the presented effective *T* energy gap (Fig. 6) can be considered as its relevant estimate.

Above, we had assumed the low-energy PL peak at ~0.85 eV to be due to the optical transitions which are taking place in Bi nanocrystals with diameters of about 10 nm. The presented calculations for the *d* = 10 nm QDs predict the *E*_g,eff_ = 0.76 eV effective energy gap, which is in a reasonable agreement with the experiment and, therefore, supports the hypothetical assumption of the low-energy PL peak origin.

## Conclusions

In summary, multiple GaAsBi/AlAs-layered quantum-well structures were grown by a mixed MBE/MEE process on GaAs substrates. After post-growth thermal annealing of the structures at 750 °C, numerous relatively low-dispersed nanoparticles were nucleated within GaAsBi quantum wells. HRTEM, EDS, and Raman spectroscopy measurements show that the nanocrystals are predominantly composed of bismuth. The photoluminescence measurements carried out reveal an additional low-energy, ≈0.85 eV, PL peak which appears in the annealed samples. The low-energy PL peak presumably can be due to optical transitions in Bi nanocrystals, which by the quantum size effects are transformed to the direct-bandgap semiconducting state. The carried out estimates of the Bi quantum dots energy spectrum support the assumption. Further and more detailed experimental and theoretical work is required for a definite answer.

## Abbreviations

EDS: Energy-dispersive spectroscopy
HAADF: High-angle annular dark-field
HRTEM: High-resolution transmission electron microscopy
MBE: Molecular beam epitaxy
MEE: Migration-enhanced epitaxy
PL: Photoluminescence
QD: Quantum dot
QW: Quantum well
RTA: Rapid thermal annealing
STEM: Scanning transmission electron microscopy
TO and LO: Transverse optical and longitudinal optical phonon modes, respectively
